# Effectiveness of tinnitus therapy using a mobile application

**DOI:** 10.1007/s00405-021-06767-9

**Published:** 2021-03-30

**Authors:** Justyna Kutyba, Elżbieta Gos, Wiesław Wiktor Jędrzejczak, Danuta Raj-Koziak, Lucyna Karpiesz, Iwona Niedziałek, Henryk Skarżyński, Piotr Henryk Skarżyński

**Affiliations:** 1 World Hearing Center, Institute of Physiology and Pathology of Hearing, Mokra 17 Street, 05-830 Warsaw/Kajetany, Poland; 2 Heart Failure and Cardiac Rehabilitation Department, Faculty of Medicine, Medical University of Warsaw, Warszawa, Poland; 3 Institute of Sensory Organs, Nadarzyn/Kajetany, Poland

**Keywords:** Mobile applications, Tinnitus, Sound therapy, telemedicine

## Abstract

**Background:**

The World Health Organization reports that the number of tinnitus sufferers is increasing year on year. Given the common use of mobile devices and the availability of applications designed to support patients in tinnitus therapy and reduce tinnitus severity, patients seeking help are likely to try this form of support. The aim of this study was to evaluate the effectiveness of a mobile application in tinnitus sound therapy, in this case ReSound Tinnitus Relief™.

**Methods:**

The study involved 52 patients hospitalized for tinnitus. All participants used the free ReSound Tinnitus Relief application for 6 months. The application is based on sound therapy. Patients were advised to use the application for at least 30 min per day, the sounds should not completely mask the tinnitus, and they should be listened to via a loudspeaker. The effects of the therapy were evaluated by means of standardized questionnaires for tinnitus severity: the Tinnitus Handicap Inventory and the Tinnitus Functional Index.

**Results:**

The study showed a reduction in tinnitus severity as measured by both questionnaires. The general severity decreased after the first 3 months and again in the following 3 months of using the application. In both questionnaires the biggest changes were observed in the subscales of emotions.

**Conclusions:**

Results obtained here from standardized questionnaires indicate that the tested application may contribute to tinnitus reduction. However, it is advisable to conduct further research on the applicability of such technology in medical practice.

## Introduction

Today, modern technologies are used in all areas of life. Patients are increasingly willing to use unconventional methods of treatment and innovative approaches to problems. Due to a growing interest in new technologies, the developers of mobile applications have created a number of tools that can be useful in medicine, for example for diabetics [1] or asthmatics [2]. There are also applications in audiology, such as noise meters, hearing screening applications, or applications to help treat tinnitus [3].

Subjective tinnitus is an internal sound perceived only by the person themselves [4]. It can be described as a squeak, noise, rumble, humming, buzzing, or crackling. People with tinnitus experience the sound as coming from one or both ears or “in the head” [4]. The presence of tinnitus can have a negative impact on the overall functioning of a patient in their private and professional environment. The phenomenon may give rise to difficulties in concentration, a tendency to fall asleep, and mental disorders such as depression, anxiety, and general irritation [5]. Tinnitus becomes annoying in situations, where the patient cannot accept it, negatively affecting their general quality of life as well as their family [6]. Tinnitus occurs in a large fraction of the population (5–20%) and its incidence increases with age [7, 8]. A recent World Health Organization report suggests there may soon be a significant increase in the population who experiences tinnitus and there will be a growing demand for effective treatments [9].

So far, there is no fully satisfactory and effective therapy for tinnitus. There are no approved pharmaceutical treatments for tinnitus, and patients often use off-label drugs (e.g., anti-vertigo products, antidepressants, sedatives, gingko-biloba extract). Hall et al. [10] showed that most of general practitioners and ENT specialists (> 60%) were dissatisfied with current drug treatments. A meta-analysis of randomized controlled trials revealed some benefits of cognitive behavioral therapy—a medium effect (0.54) for therapist-delivered CBT and a small effect (0.38) for internet-delivered CBT [11]. Wang et al. [12] in their systematic review of sound therapy concluded that it can suppress tinnitus in some patients, but there is still limited evidence of its effectiveness. Currently, when organic disease has been excluded, the standard health care procedure is to provide the patient with sound therapy, cognitive–behavioral therapy, or relaxation therapy [4]. Moreover, sound therapy is often self-help in the hope of alleviating the nuisance of tinnitus for a long time (habituation) or even for a short time (masking).

Sound therapy is a broad term that can be used in many ways. According to the American Tinnitus Association, it is the use of an external acoustic signal to change the perception of tinnitus or one’s reaction to it [13]. This form of therapy does not cure tinnitus, but can significantly reduce the annoyance and negative reactions caused by it [14]. Four mechanisms of sound therapy can be distinguished: masking, distraction, habituation, and neuromodulation [15]. Current clinical practice in our clinic is to a large extent aimed at habituation to tinnitus, i.e., the use of an external signal to reclassify tinnitus as an insignificant sound that the patient should subconsciously ignore. Sound therapy may use various devices, including sound generators, broadband noise generators, or hearing aids [6].

The main rationale for the present study is that, as a result of the common use of mobile devices, mobile applications have been gaining more popularity among tinnitus patients. Mobile applications are tools installed on mobile phones whose principles of operation are practically identical to those of conventional generators. The creators of mobile applications all claim these tools meet their objectives. Unfortunately there is still a lack of research confirming their effectiveness [16, 17]. In March 2016, when this study began, more than 200 tinnitus applications were available on “Google Play” and “App Store” [18]. Although there are a few studies that have evaluated the effectiveness of mobile applications for tinnitus, they are usually based on a very small number of subjects and lack longitudinal outcomes [19, 20]. Another consideration was that, based on the assumption that it is the sound itself which is important, not the device that emits it, one can assume that sound therapy using mobile applications should be as effective as using a classical sound generator. Moreover, findings from one application might be useful for improving others [21, 22].

We, therefore, picked one well-featured and representative mobile application for sound therapy—ReSound Tinnitus Relief. The objective of the current study was to assess its effectiveness over half a year.

## Materials and methods

### Participants and setting

The study was approved by the Bioethics Committee of the Institute of Physiology and Pathology of Hearing (No. KB/03.2017). The participants were adults who in 2017–18 had been hospitalized for tinnitus at the Institute of Physiology and Pathology of Hearing. In Poland, patients have the opportunity to be admitted to an audiological ward for diagnosis of tinnitus. During a 3 day stay in the facility, a set of audiological examinations is performed and the patient undergoes medical, psychological, and audioprosthetic consultations.

The participants of this study gave written agreement to participate and for their data to be used for scientific purposes. The recruited persons met the criteria set out in Table 1.

**Table 1:** Inclusion and exclusion criteria for subjects

Inclusion criteria	Exclusion criteria
- Adults - Annoying, continuous tinnitus - Subjective tinnitus - Physically and mentally fit to the extent that they are able to fill in the questionnaires themselves - Persons who have a mobile phone with android or ios	- Children and people under 18 years of age - Intermittent tinnitus - Objective tinnitus - Severe or profound hearing loss - No device enabling the use of the application - Disability preventing them from completing the questionnaires themselves

Initially, 96 persons qualified for the study; however, 44 were excluded due to 3 reasons:

- lack of complete data—questionnaires incomplete and not suitable for analysis.
- discontinuation of use of the application before the end of the study—a problem with accepting sound therapy.
- loss of contact—no response to mail communication.

Finally, results obtained from 52 patients were analyzed, made up of 27 (52%) women and 25 (48%) men. The average age of participants was 48 years (SD = 13.8), with range from 18 to 73 years. The participants mostly had higher or secondary education (94%) and lived in cities with more than 200,000 inhabitants (45%). More than half the participants (61.5%) experienced tinnitus in both ears, while the remaining 38.5% reported it came from one ear only. The mean duration of tinnitus was 5.4 years (SD = 5.9) and ranged from 1 to 30 years. Pure tone average (PTA) hearing threshold for air conduction was 20.0 dB (SD = 12.7) for the right ear and 20.8 dB (SD = 11.8) for the left. For bone conduction, PTA was 16.0 dB (SD = 12.3) for the right ear and 16.3 dB (SD = 11.5) for the left. From the medical interview, 31% of subjects indicated they suffered dizziness and 25% of them had a balance problem.

Among those recruited for the study, 46% had used other forms of therapy before their visit to the Institute of Physiology and Pathology of Hearing. In most cases it was pharmacotherapy (87%). Other methods included sound masking, cognitive–behavioral therapy, and oxygen hyperbaric chambers. In the opinion of the patients, none of these methods had affected the level of their tinnitus.

### ReSound Tinnitus Relief app

**Fig. 1:**
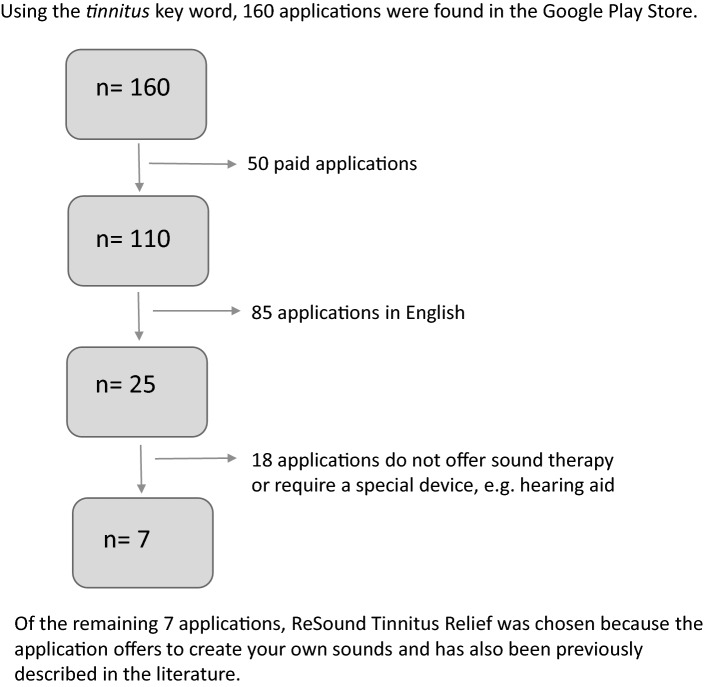
Selection process of application

From 200 applications available from Google Play and the App Store, we limited the selection to 160 from Google Play. Using the criteria of Table 2, we conducted a further selection process. Figure 1 shows the application selection process. First, paid applications were excluded, then we chose applications in Polish, those which offered sound therapy, and finally did not require any additional device. In this way, 7 applications were selected: ReSound Tinnitus Relief, Beltone Tinnitus Calmer, Tinnitus Synthesizer, Tinnitus relief app Sound Therapy, Biały szum i dźwięki snu, Odgłosy natury, and Biały szum—Sound Relaxing. From this group the application developed by ReSound (ReSound Tinnitus Relief version 5.2.0) was selected [23]. This application not only met all the criteria but has also been previously described in the literature. This made it possible to compare our results with those obtained previously. The main aim of ReSound is to help patients by reducing the contrast between perceived tinnitus and ambient sounds, thereby reducing severity. This is achieved using an extensive library of sounds from which the patient chooses suitable ones as stimuli. Sounds are divided into three categories: environmental, music, and therapeutic sounds.

**Table 2:** Criteria for selecting a tested application

Inclusion criteria	Exclusion criteria
- Free of charge - In Polish - Using elements of sound therapy - Enables patients to have more options than a conventional sound generator - Supported by phones with Android or IOS systems	- Chargeable - In a language other than Polish - Mechanism of action not based on sound therapy - Requiring a special device (other than a mobile phone) - Special software requirements

The following therapeutic recommendations were given to the participants on how they should use the application:

- sound selection—the participants had to decide themselves which sounds they wanted to listen to.
- time—the participants themselves had to decide when to use the application and for how long, but no less than 30 min per day. The patient simply adjusted the time of use to suit their individual routines. They were advised to use the application when tinnitus was very loud and disrupted their daily tasks, when they were in a silent environment, and before bedtime.
- the way of listening to the sounds—generally listen from the phone’s loudspeaker (free-field), but if necessary headphones could be used. The sounds should create an acoustic background; that is, they should be played spatially, just like bedside generators.
- the level of the emitted signal—the sounds emitted should not completely mask the patient’s tinnitus but should be slightly quieter. The sounds were supposed to enrich the acoustic background, not mask the tinnitus. In such a situation, tinnitus will break through the acoustic background and be audible, but the contrast between the tinnitus and the acoustic background is small. When tinnitus occurs in the presence of additional ambient sound, it is no longer the main acoustic stimulus for the brain. This speeds up the process of habituation to tinnitus [21, 24, 25].

All persons participating in the study were able to contact an audiologist by phone.

### Measures

Standardized questionnaires were used to evaluate the effects of the therapy:

- Tinnitus Functional Index (TFI)—a questionnaire used to assess treatment-related changes in tinnitus and for scaling its overall severity [26]. The questionnaire consists of 25 questions and evaluates the patient’s functioning on 8 subscales: intrusiveness, sense of control, cognition, sleep, auditory, relaxation, quality of life, and emotional distress. The scores for each question range from 0 to 10. The patient can obtain a maximum of 100 points, with the higher the score, the greater the severity of the ailment. An improvement of at least 13 points in total score reflects a clinically important change in tinnitus severity, as proposed by the authors of TFI [27]. The Polish version of the TFI questionnaire was licensed from the Oregon Health & Science University for use in clinical and scientific research. Its psychometric properties were comparable to the original version (its factor structure has been confirmed; its internal consistency is very high with a Cronbach’s alpha coefficient of 0.96; convergent and discriminant validities have also been confirmed) [28].
- Tinnitus Handicap Inventory (THI)—a questionnaire for a self-reported measure of the impact of tinnitus on daily living. It focuses on 3 subscales: functional, emotional, and catastrophic reactions [29]. The patient chooses one of three answers: yes, sometimes, and no. The maximum score is 100 points, with the higher the score, the more severe the discomfort. An improvement of 20 points or more in total score is treated as a clinically important change in tinnitus severity, according to Newman et al. [30]. Psychometric properties of the Polish version of the THI have been documented by Skarzynski et al. [31]. A Cronbach’s alpha coefficient of 0.95 indicates very high internal consistency, and its convergent validity has been confirmed with a strong correlation (*r* = 0.75) with another tinnitus questionnaire (the Tinnitus and Hearing Survey). In 2020 normative values for THI were established for a Polish population of tinnitus sufferers; the values can be used as a benchmark in clinical practice and scientific research [32].
- Application usage assessment survey—created for the purposes of this study. Questions included in the survey concerned the quality of use of the application and the type of sounds listened to.

### Patient assessment procedure

The research was conducted according to the following protocol.

First visit:

- patient recruited during last day of 3 day hospitalization for tinnitus;
- the nature and purpose of the research explained; informed consent form signed;
- filling in of TFI and THI;
- participation in a group meeting which covered:
  o information on sound therapy and how it can affect auditory function
  p the capabilities of the application and the way it operates;
- installation of the application on the patient’s mobile phone.

After starting to use the application, 3 and 6 months later the participants received through the mail another set of TFI and THI questionnaires and an application usage assessment survey. The patients were asked to fill in the questionnaires and send them back.

### Statistical analysis

Descriptive statistics for THI and TFI scores were calculated. To evaluate differences in tinnitus severity perceived by the patients over the three measurement times (baseline, 3 months, and 6 months), a repeated measures ANOVA with Bonferroni correction was applied. There were 13 variables which were compared at 3 measurement points, so to control for the familywise error rate the *p*-level used was 0.05/39 = 0.00128. For statistical analysis, IBM SPSS Statistics v. 24 software was used.

## Results

### Changes in tinnitus severity as measured with THI

The initial average level of tinnitus severity as perceived by the patients and measured with the THI global score was 54.4 (SD = 22.1); after 3 months of using ReSound it decreased to 47.9 (SD = 22.7); and after 6 months it was 35.0 (SD = 19.8). The effect was statistically significant: *F*(2102) = 62.4; *p* < 0.00128; *e*^2^ = 0.550 and multiple comparisons showed that tinnitus severity at 6 month follow-up had significantly decreased in comparison to baseline and to 3 month follow-up, but there was no statistically significant difference between 3 month follow-up and baseline). A similar effect was observed for the Functional subscale: *F *(2102) = 44.2; *p* < 0.00128; *e*^2^ = 0.465.

Statistically significant differences were revealed for the Emotional and Catastrophic subscales: *F*(2102) = 40.51; *p* < 0.00128; *e*^2^ = 0.443 and *F*(2102) = 43.04; *p* < 0.00128; *e*^2^ = 0.456, respectively. Multiple comparisons showed that tinnitus severity in each follow-up had significantly decreased.

Descriptive statistics for all THI subscales and global score are presented in Table 3.

**Table 3:** Descriptive statistics of the results of tinnitus severity measured with THI

THI subscale	Period	Min	Max	M	SD	Q1	Me	Q3
Functional	Baseline	2	44	24.35	10.65	16.50	26.00	32.00
	3 month follow-up	2	42	22.63	10.61	12.50	23.00	30.00
	6 month follow-up	0	38	16.77	9.95	6.50	18.00	22.00
Emotional	Baseline	0	36	18.21	8.68	12.00	17.00	26.00
	3 month follow-up	0	36	15.65	9.10	8.50	16.00	22.00
	6 month follow-up	0	26	10.77	7.47	4.00	12.00	17.50
Catastrophic	Baseline	2	20	11.85	4.41	10.00	12.00	14.00
	3 month follow-up	0	20	9.63	4.94	6.00	10.00	13.50
	6 month follow-up	0	20	7.42	4.34	4.00	7.00	10.00
THI global	Baseline	6	100	54.40	22.09	38.25	55.00	71.50
	3 month follow-up	10	96	47.92	22.73	30.00	48.00	67.50
	6 month follow-up	0	72	34.96	19.84	16.50	36.00	50.00

*M* mean, *SD* standard deviation, *Q1* lower quartile, *Me* median, *Q3* upper quartile

Clinically significant improvement in THI was revealed in 6 patients (11.5%) at the 3 month follow-up and in 28 patients (53.8%) at the 6 month follow-up.

### Changes in tinnitus severity measured with TFI

The average level of tinnitus severity as measured with the TFI global score at the baseline was 51.1 (SD = 22.8); after 3 months of using ReSound it decreased to 44.8 (SD = 20.9); and after 6 months, it was 36.8 (SD = 19.0). The effect was statistically significant: *F*(2,102) = 28.9; *p* < 0.00128; *e*^2^ = 0.362 and multiple comparisons showed that tinnitus severity at 6 month follow-up had significantly decreased in comparison to baseline and to 3 month follow-up, but there was no statistically significant difference between 3 month follow-up and baseline).

Analysis showed that results for almost all TFI subscales were statistically significant, the only exception being the Cognition subscale.

The biggest effect was revealed for the Emotional subscale: *F*(2102) = 23.1; *p* < 0.00128; *e*^2^ = 0.311. Multiple comparisons showed that problems with emotions significantly decreased at 3 month follow-up compared to baseline and at 6 month follow-up compared to 3 month follow-up, but there was no statistically significant difference between 6- and 3 month follow-up.

The effect for Sense of Control was *F*(2,102) = 9.1; *p* < 0.00128; *e*^2^ = 0.152. Problems with sense of control were significantly lower at 6 months than at baseline, but it was stable between 3 months and baseline and between 6 and 3 months.

For other subscales the following results were obtained: Intrusiveness: *F*(2102) = 19.7; *p* < 0.00128; *e*^2^ = 0.278; Sleep: *F*(2102) = 17.6; *p* < 0.00128; *e*^2^ = 0.257; Auditory: *F*(2102) = 14.6; *p* < 0.00128; *e*^2^ = 0.222; Relaxation: *F*(2,102) = 17.1; *p* < 0.00128; *e*^2^ = 0.251; Quality of life: *F*(2102) = 19.9; *p* < 0.00128; *e*^2^ = 0.281. In these aspects of tinnitus, results at the 6 month follow-up were lower than at baseline, while no differences between baseline and 3 months were observed.

**Fig. 2:**
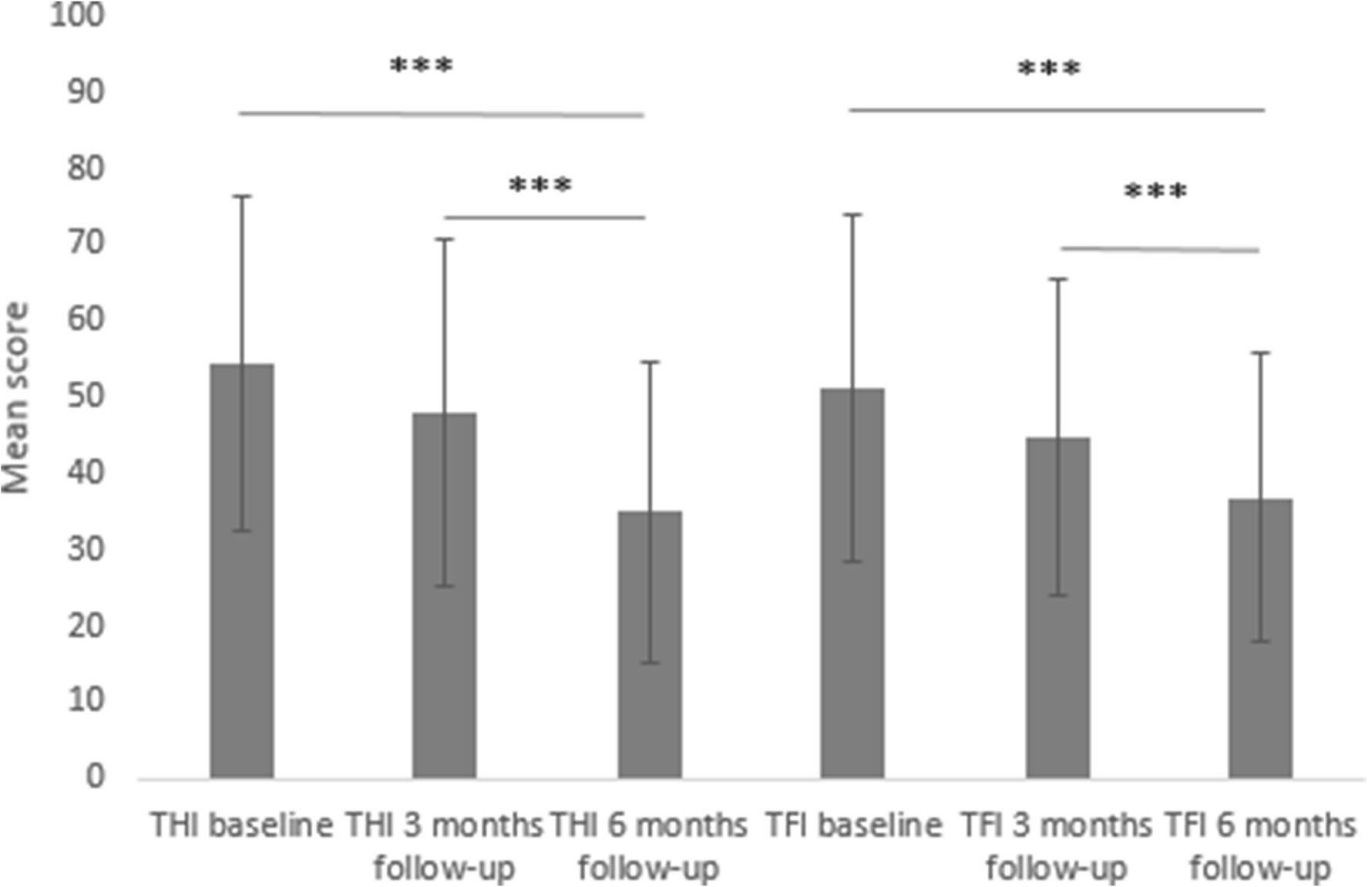
Average results of THI and TFI global scores. The error bars are standard deviations. ***p < 0.001

Descriptive statistics for all TFI subscales and global score are presented in Table 4. The differences in tinnitus severity as measured by THI and TFI global scores are summarized in Fig. 2.

**Table 4:** Descriptive statistics of the results of tinnitus severity measured with TFI

TFI subscale	Period	Min	Max	M	SD	Q1	Me	Q3
Intrusiveness	Baseline	20.00	100.00	64.74	21.53	50.83	68.33	80.00
	3 month follow-up	0.00	100.00	58.40	22.87	40.00	63.33	76.67
	6 month follow-up	10.00	86.67	49.10	22.20	26.67	50.00	69.17
Sense of Control	Baseline	6.67	100.00	48.08	26.86	23.33	48.33	70.00
	3 month follow-up	0.00	90.00	40.64	23.14	20.00	38.33	56.67
	6 month follow-up	0.00	86.67	35.71	21.98	13.33	36.67	56.67
Cognition	Baseline	0.00	100.00	44.17	27.30	20.83	46.67	60.00
	3 month follow-up	0.00	90.00	40.45	23.90	20.00	40.00	56.67
	6 month follow-up	0.00	80.00	34.55	22.56	16.67	30.00	50.00
Sleep	Baseline	3.33	100.00	55.32	30.13	30.83	51.67	80.00
	3 month follow-up	0.00	100.00	51.03	27.61	30.83	48.33	73.33
	6 month follow-up	0.00	90.00	41.99	23.86	26.67	38.33	63.33
Auditory	Baseline	0.00	100.00	49.68	27.80	26.67	58.33	70.00
	3 month follow-up	0.00	100.00	43.91	24.66	26.67	50.00	56.67
	6 month follow-up	0.00	86.67	36.28	22.24	20.83	33.33	50.00
Relaxation	Baseline	6.67	100.00	55.51	28.39	33.33	56.67	75.00
	3 month follow-up	0.00	100.00	49.68	26.59	30.00	45.00	75.00
	6 month follow-up	0.00	83.33	39.81	24.55	20.00	38.33	60.00
Quality of Life	Baseline	0.00	100.00	45.67	28.66	23.13	43.75	70.00
	3 month follow-up	0.00	100.00	39.81	27.29	15.00	41.25	60.00
	6 month follow-up	0.00	80.00	29.52	23.38	10.00	30.00	44.38
Emotional	Baseline	6.67	100.00	47.31	26.99	20.00	50.00	69.17
	3 month follow-up	0.00	96.67	36.03	26.56	14.67	30.00	60.00
	6 month follow-up	0.00	80.00	29.62	23.84	7.50	23.33	46.67
Total	Baseline	8.80	96.40	51.08	22.80	35.20	51.60	66.80
	3 month follow-up	0.80	95.60	44.78	20.91	29.50	42.60	60.20
	6 month follow-up	1.20	72.80	36.77	18.99	20.70	35.00	47.80

*M* mean, *SD* standard deviation, *Q1* lower quartile, *Me* median, *Q3* upper quartile

As can be seen in Fig. 2, tinnitus severity, measured with both the THI and TFI global scores, significantly decreased over the 6 month period of using the mobile application. It was noticed in particular at the 6 month follow-up in comparison to baseline and at the 3 month follow-up.

Clinically significant improvement in TFI was found in 14 patients (27%) at the 3 month follow-up and in 28 patients (58%) at the 6 month follow-up.

### Application usage assessment survey

Data obtained from the survey showed that for 86.4% of users, the application was very easy or easy to use. Most people (84.6%) also said that the sounds presented sounded natural. More than 73% of people stated that they liked to use the app and used it daily, and 76.8% expressed their willingness to use the app when the study ended.

The sounds most frequently chosen by the study participants were:

- ocean surf—46%
- relaxing music—37%
- birds—27%
- rain—25%.

**Fig. 3:**
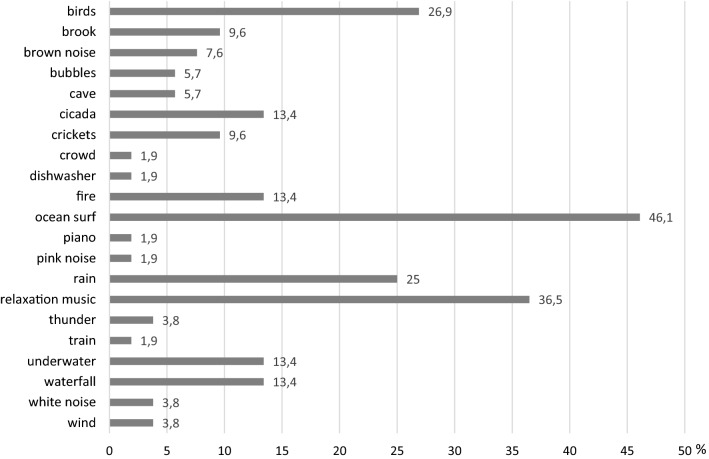
Sounds listened to by participants

The exact data concerning the sounds listened to can be found in Fig. 3.

### Characteristics of the excluded patients

There were 44 dropouts, 34% women and 65% men. Their averaged age was 46 years (SD = 12.3), with range from 21 to 71 years. Some 58% of them experienced tinnitus in both ears, while the remaining 42% in one ear. The mean duration of tinnitus was 5.2 years (SD = 5.3) and ranged from 0.5 to 21 years. There were 91% of the dropouts who had had a higher or secondary education, 32% lived in cities with more than 200,000 inhabitants, 36% experienced dizziness, and 22% had a balance problem.

Air conduction PTA was 22.8 dB (SD = 16.5) for the right ears and 20.0 dB (SD = 15.1) for the left. For bone conduction, PTA was 18.2 dB (SD = 16.5) for the right ears and 16.2 dB (SD = 15.2) for the left.

For 32 dropouts we had THI and TFI scores. The initial level of tinnitus severity measured with the THI global score ranged from 6 to 82 points and the mean was 41.4 (SD = 19.6). For TFI it was ranged from 8.4 to 69.2 points with a mean of 35.6 (SD = 14.9).

In brief, the sociodemographic characteristics were similar in the participants and in the dropouts (except that there were more men in the dropouts than in the study group). Tinnitus severity was much lower in the excluded patients than in the included patients.

## Discussion

The current study shows that continuous use of sound therapy for half a year provided by the mobile application ReSound Tinnitus Relief coincided with a decrease in tinnitus symptoms as measured by THI and TFI. Both questionnaires showed that the biggest changes were in emotional status. This may not be surprising, since emotional and psychological problems are known to go side by side with tinnitus [33, 34]. Studies published so far concerning conventional sound therapy using a sound generator (SG) have shown that such devices can relieve the severity of tinnitus. Rocha et al. [35] showed on a group of 30 patients that SG can be helpful both for people with isolated tinnitus and people with tinnitus and hearing loss. At the 6 month follow-up, the overall results of the THI questionnaire were reduced in the group of people with tinnitus and normal hearing from M = 66.7 (SD = 12.3) to M = 11.6 (SD = 10.0), and in the group with tinnitus and hearing loss, from M = 66.4 (SD = 13.8) to M = 10.1 (SD = 12.9). The observed changes were statistically significant. Another study of 10 people by Suzuki et al. [36] also showed a reduction in the severity of tinnitus as measured by the THI questionnaire. A reduction of at least 20 points was found in 9 people, but due to the small group this result was not statistically significant. In both the above-mentioned studies, behind the ear (BTE) SGs were used, which could have affected the results, because the sounds were emitted through the earphones directly into the external ear canal. The use of sound generators shows promising results and it might encourage patients to choose these devices. However, in compare with mobile applications which are mostly free and easy available, the sound generators are expensive devices. The cost may be the important difference between the sound generator and the mobile application.

On the other hand, a systematic review by Sereda et al. [37] showed that there was no evidence that one type of sound therapy device (i.e., hearing aid, SG, or combination hearing aid) was more effective than the other. Our study showed that there was a statistically significant difference in the perception of tinnitus after the use of sound therapy via a mobile application. Mobile applications are more flexible devices than sound generators. Thanks to the ability to update and modify a mobile application, it is easier for patients to customize the tool to their individual needs. Greater availability and low cost (most mobile apps are free) can also help achieve better results. A wide selection of free apps allows the user to experiment with each, so the patient can choose the best one for their own needs.

There are only a few studies on the use of mobile applications in tinnitus therapy [38]. For example, Henry et al. [19] developed one and tested it on a group of 25 people for 8 weeks. The recorded general changes in the average TFI results were small, but almost one-third of the users (8 out of 25), obtained a reduction of 13 points or more. According to the authors of the TFI questionnaire, a decrease of 13 points or more reflects a change that may be significant for a given person. The sounds best rated by the participants of this study were: ocean waves, rain, fan, and frogs. Mehta et al. [39] used standardized tools (THI questionnaire and a visual analog scale) to assess the impact of the HyperSound tinnitus application. According to a visual analog scale, a significant reduction in loudness and severity of perceived tinnitus was obtained. The THI questionnaire showed a decrease in overall mean score from 43 (SD = 30) to 39 (SD = 29), but these results were not statistically significant (*p* = 0.06), probably caused by the small sample size (*n* = 11). The observation time was very short (5 min) and there was no information about the long-term effects of the therapy.

There are also distinct groups of tinnitus sufferers who have profound hearing loss in one ear (called single-sided deafness, SSD) or in both ears. Cochlear implants (CIs) prove to be a very effective treatment which restores their ability to hear [40]. There is also the side-effect that a CI reduces tinnitus in around 90% of subjects and even eliminates it in around 20% [41–44]. These numbers might be improved even further, and therefore, CI users might also be potential users of mobile applications for tinnitus. In fact, Tyler and colleagues used the same app as the one in the current study (ReSound) on CI subjects [20]. The investigation focused mainly on the acceptability of the presented sounds. According to the CI users, the most acceptable sound was rain and waves on rocks. For 90% of study participants the therapy was satisfactory, but only 16 people took part. It was also found that using an application on a mobile phone was more convenient for patients than using tablets or computers. The participants said that the application "provided them with a wide variety of sounds" and the signals provided by the implant were acceptable.

The studies above describe preliminary tests of mobile applications and innovative devices used today to reduce tinnitus severity. The results might be generally classed as satisfactory, although it is not possible to compare them accurately because of small samples and different methodologies. In our study we found a decrease in tinnitus severity as measured with the THI questionnaire. General severity slightly decreased after 3 months of sound therapy with the ReSound application and it decreased significantly after another 3 months. This change was particularly apparent in the negative emotions associated with tinnitus. Many researchers emphasize that tinnitus is associated with lowered mood, irritability, and increased stress [45, 46]. In this context, the results of this study can be considered particularly encouraging. Our research showed that reduction of negative emotions occurred faster than in the reduction of the Functional subscale, where change was only noticeable after 6 months. The Functional subscale concerns aspects such as concentration, sleep problems, limiting social contacts due to tinnitus, and others—i.e., cognitive strategies and patterns of behavior that are more difficult to change than emotions and so require more time. Clinically important change (improvement) in tinnitus severity measured with the THI was found in 12% and 54% of the participants after 3 and 6 months, respectively. The effect seems quite encouraging when compared, e.g., to the effect of an intervention combining counselling and hearing devices shown by Gudex et al. [47]. After 1 month of intervention it was found that 27% of the tinnitus subjects showed a clinically important change on the THI and 24% after 2 years.

Similarly to THI, a reduction in tinnitus severity was also demonstrated by TFI, where the overall rating slightly decreased after 3 months and decreased significantly after another 3 months. It is worth noting that the biggest changes were recorded in negative emotions, which is consistent with the THI results and increases the reliability of the results. The sense of control over tinnitus increased significantly after just 3 months and remained stable. Psychologists emphasize that the sense of control over health is an important factor [34, 48]. It seems particularly beneficial for tinnitus patients to have a tool by which they can readily relieve themselves of chronic and troublesome discomfort. Tinnitus control by means of an external agent has a positive effect on self-empowerment and improves the psychological functioning of the sufferer. We found clinically important improvement in tinnitus severity measured with the TFI in 27% of the participants after 3 months and in 58% of the participants after 6 months. The effect was comparable or even better than that revealed by Jacquemin et al. [49]. In this study authors found that the improvement in tinnitus treatment (High-definition transcranial direct current stimulation) was significant for 50% of the responders who underwent 6 sessions of neuromodulation 7 weeks earlier. So, our result is comparable and serves as additional evidence for the effectiveness of tinnitus therapy using a mobile application.

As in the case of THI, TFI also showed beneficial (although more delayed) effects of the sound therapy on functional aspects of tinnitus such as cognition, sleep, hearing, and relaxation. For these aspects, it was necessary to wait over 3 months for positive effects, which also indicates the need for careful planning of mobile device therapy and to convince the patient that systematic and long-term use of the application is needed.

### Limitations

In this work, similarly to other earlier works, the main limitation is the lack of a control group [19, 20]. There was also a lack of objective measurement of the time the application was used. Unfortunately the application did not provide the opportunity to gain such data. The results of the TFI and THI questionnaires indicated a reduction in annoyance, but it is not known whether a similar effect could be achieved without using any form of therapy. When starting this study we had a dilemma: should we try to convince some patients to refrain from using the application when they wanted to? We decided this would not be ethical. At the same time, we failed to convince some patients who did not intend to use the application to fill in the TFI and THI questionnaires within the required time periods. Also the participants did not report whether they used other forms of therapy during the experiment. It is, therefore, difficult to determine the extent to which these additional factors affected the results.

## Conclusions

This study involves the largest number of people in terms of published studies to date. The results from standardized questionnaires from a group of 52 patients indicate that the tested application may contribute to tinnitus reduction. However, to be able to draw more certain conclusions, the study needs to be conducted in a more controlled environment. The questions which arose during analysis of the data are a catalyst for improving the protocol. The current study is being continued, and now includes a control group and more accurate monitoring of the experimental group.
